# Supplementary material for: Cold shock induces a terminal investment reproductive response in C. elegans
Source: Sci Rep. 2022 Jan 25;12:1338. doi: 10.1038/s41598-022-05340-6 (PMC8789813; doi:10.1038/s41598-022-05340-6)
Supplement: Supplementary file 1 — Supplementary Information. [file 41598_2022_5340_MOESM1_ESM.docx]

**Supplemental Figures**

| 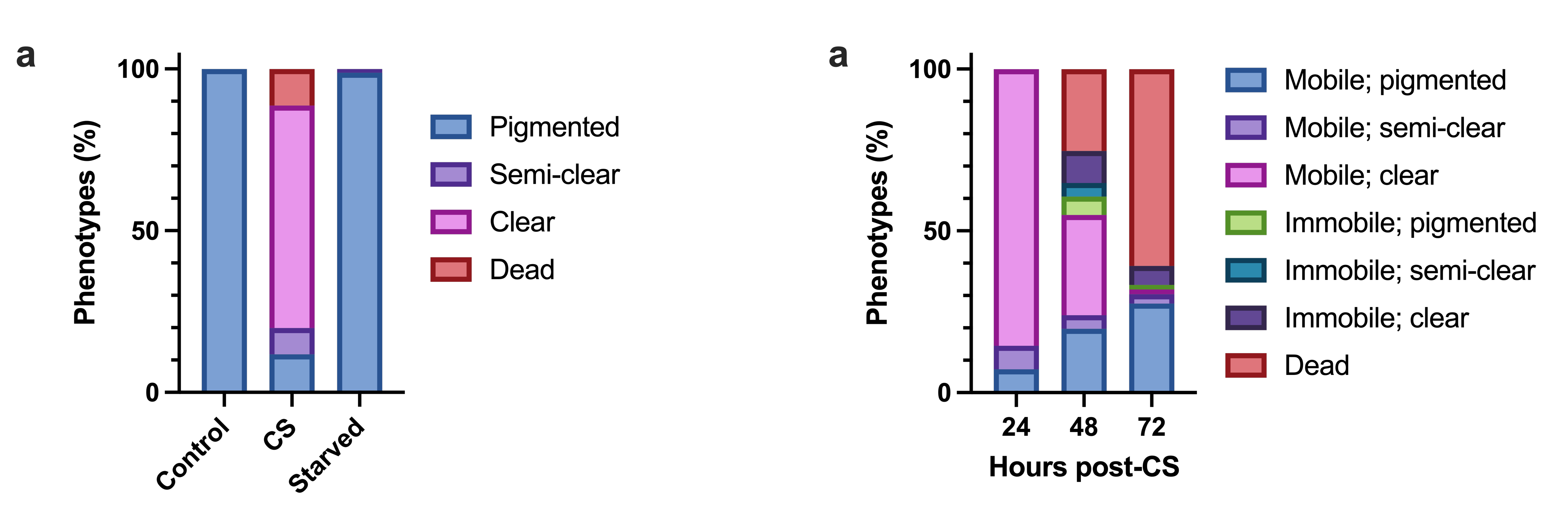 |
| --- |
| **Supplemental Figure S1. Cold shock-induced phenotypic changes do not result from starvation during shock.** N2 young adult hermaphrodites on seeded (*E.coli* OP50 food source) plates were shifted from 20°C to 2°C for a 4 h cold shock (CS) or mock shock, while worms on plates lacking a food source were starved for 4 h prior to food reintroduction. Worms then recovered at 20°C for 24 h with assessment of phenotypic alterations. Chi-squared Test for Homogeneity: P < 0.0001 at 24 h NS CS vs. starved (n = 459 N2 control; n ≥ 960 worms for all other conditions). |

| 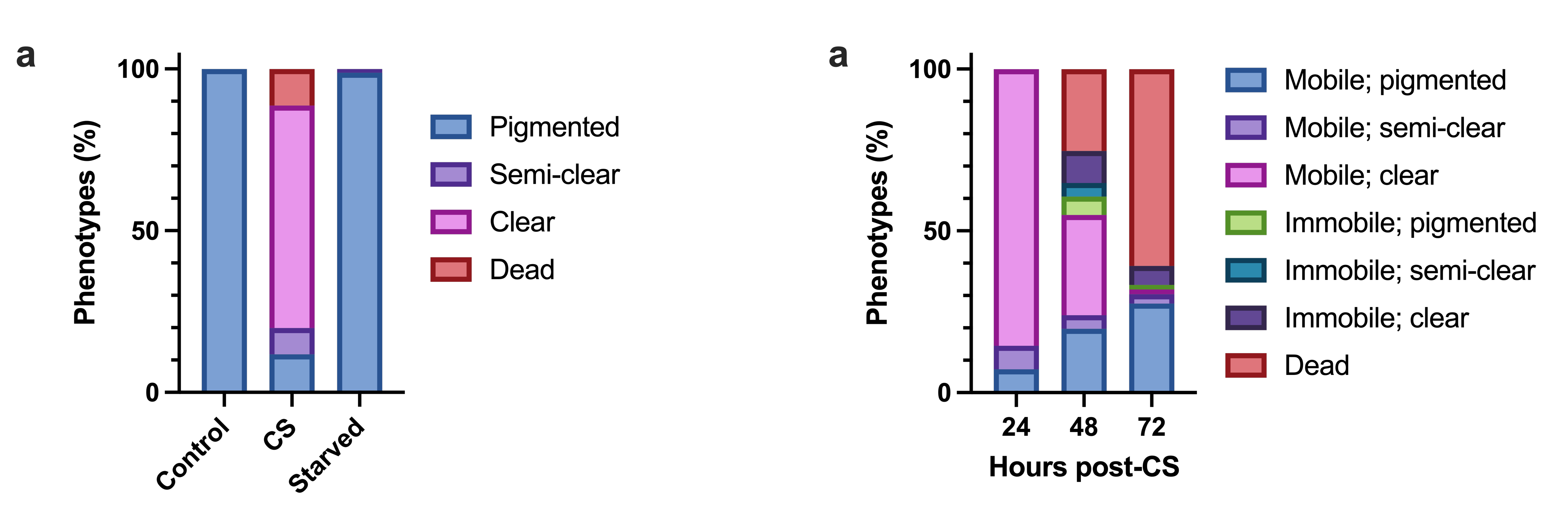 |
| --- |
| **Supplemental Figure S2. *skn-1(zj15)* loss-of-function mutants show comparable phenotypic transitions to other *skn-1* loss-of-function alleles.** Young adult hermaphrodite *skn-1(zj15*) loss-of-function mutants were shifted from 20°C to 2°C for a 4 h CS and then recovered at 20°C for 72 h with assessment of phenotypic alterations (n = 69). |
